# Much more than cigarette smoking

**DOI:** 10.1590/S1806-37132014000200001

**Published:** 2014

**Authors:** Jaqueline Scholz Issa, Gabriel Magalhães Lopes

**Affiliations:** Smoking Cessation Program, Department of Cardiology, Heart Institute, University of São Paulo School of Medicine Hospital das Clínicas, São Paulo, Brazil; Division of Childhood and Adolescent Psychiatry and Balance Program, Department and Institute of Psychiatry, University of São Paulo School of Medicine Hospital das Clínicas, São Paulo, Brazil

Cardiologist in Charge, Smoking Cessation Program, Department of Cardiology, Heart Institute, University of São Paulo School of Medicine Hospital das Clínicas, São Paulo, Brazil Psychiatrist, Division of Childhood and Adolescent Psychiatry and Balance Program, Department and Institute of Psychiatry, University of São Paulo School of Medicine Hospital das Clínicas, São Paulo, Brazil

The human condition made fragile by fear of death, by affective distress, by diseases, and by aging compels human beings to search for pleasure. The use of licit or illicit drugs is a good example of this fact. Often, this occurs in such an intense way that it condemns individuals to premature death or a subjugated life. To compound this situation, Western society is continuously bombarded by advertisements that encourage the consumption of products. Consumption becomes synonymous with happiness.

The tobacco industry may have been the pioneer in exploiting and addressing this aspect in cigarette advertising,^(^ ^1^ ^)^ being responsible for inducing billions of users to dependence on the product and, consequently, to premature death and loss of quality of life, placing an unprecedented burden on health care systems worldwide. The consequence of this tobacco epidemic was the adoption of restrictive measures to reduce consumption, and this motivated the tobacco industry to search for products that would maintain nicotine dependence, despite being labeled as "less harmful to health" than typical cigarettes.^(^ ^2^ ^)^ In this context, products such as hookah or narghile, and, more recently, electronic cigarettes, began to be promoted as harmless, despite there being no scientific support for it. The evidence that there are fewer toxic substances in these alternative forms of tobacco than in conventional cigarettes has been used and exploited as an argument in support of the former being harmless. In fact, however, there are not sufficient data to establish that the use of these substances at lower concentrations, but in a continuous, endless way because of the presence of nicotine vapor, is harmless.

At the epicenter of this discussion, this issue of the Brazilian Journal of Pulmonology features an original article by Martins et al.^(^ ^3^ ^)^ on experimentation with narghile, the prevalence of its use, and the level of knowledge on the subject among third- and sixth-year medical students at the University of São Paulo School of Medicine, in the city of São Paulo, Brazil, between 2008 and 2013. Although most students recognized the potential health risk from the use of narghile, either because of the risk of contamination with bacteria and viruses or because of the very potential for nicotine dependence resulting from the use of the product, in addition to high exposure to elevated carbon monoxide levels, this knowledge did not prevent more than 40% of the male students from experimenting with it. The low prevalence of cigarette use among third- and sixth-year medical students (9.78% and 5.26%, respectively, among males, and 1.43% and 2.65%, respectively, among females) clearly shows the effectiveness of public policies discouraging smoking initiation, implemented more than 20 years ago in Brazil. On the other hand, it shows the reach of the marketing and advertising campaigns disseminating the use of alternative forms of tobacco.

The reduction in smoking in Brazil in the last 5 years has been gradual and constant among men, with smoking rates ranging from 21% in 2008 to 18% in 2012.^(^ ^4^ ^)^ Among women, smoking rates decreased and have remained stable since 2008, varying between 12% and 13%. Currently, there are more former smokers than smokers in Brazil, and they represent 22% of the population. In a systematic review of the literature on the use of alcohol and tobacco among adolescents aged 10 to 19 years, Barbosa et al.^(^ ^5^ ^)^ found that the prevalence of current tobacco use (use in the study period or in the previous month) ranged from 2.4% to 22.0%, with a mean of 9.3%, in Brazil. Although the risk of tobacco use initiation by adolescents is higher when their parents smoke, there are other family-related factors, such as poor parental practices and poor behavioral control of offspring.^(^ ^6^ ^)^ In addition to the implications of tobacco use on adolescent health, it is of note that the combined use of tobacco and marijuana is high.

The tobacco industry knows that it has factors working in its favor that greatly facilitate experimentation with any product containing nicotine among young people, including, first of all, curiosity, as well as the symbolic power of such experimentation as a rite of passage, the immaturity of brain structures involved in inhibiting impulsiveness, the influence of peers, and a desire for confrontation, that is, a set of factors that make young people particularly vulnerable to being seduced by the fascination of experimenting with and using drugs.

It is relevant to highlight the importance of cultural and media influence on young people, since adolescence is a crucial stage in the development of personality and individuality. At this stage, adolescents are very influenceable, and marketing and advertising purposely target young people. Studies have shown that cigarette advertising has a strong influence on tobacco use by young people.^(^ ^7^ ^)^ The release of formerly secret documents of the tobacco industry obtained through litigation in the USA has confirmed the industry's strategy of aiming cigarette advertising primarily at children and adolescents.

The result is that new experiences tend to be lived to the fullest and the use of alcohol, cigarettes, and other drugs is a frequent behavior, which makes this period of life crucial to most studies on and programs for prevention of substance dependence. Approximately 72% of adolescents in the USA report having experimented with alcohol; in Brazil, the prevalence is as high as 84.3% among students aged 17-18 years. ^(^ ^8^ ^)^ With regard to cigarettes, the prevalence of ever smoking among adolescents in the USA is 43.6%, and, in Brazil, the prevalence is 32.1% among 18-year-olds.

In 2009, according to the National School Heath Survey,^(^ ^9^ ^)^ 24.2% of the ninth graders had experimented with cigarettes and 6.3% smoked regularly. There were no differences between boys and girls. However, tobacco use was higher in public schools (26.4%) than in private schools (18.3%).

Environmental factors are important risk factors for experimentation with and maintenance of alcohol and tobacco use in adolescence, as well as for progression to other drugs.^(^ ^10^ ^)^ Protective factors have not been studied as extensively as have risk factors. The major protective factors against alcohol and cigarette use include ability to face and overcome problems, especially in females; religiousness; having at least one meal together as a family on most days; and parental or guardian knowledge about what adolescents have been doing in their free time in the last 30 days. Effective parental monitoring seems to be the strongest protective factor against the use of alcohol, tobacco, and other drugs in adolescence and can be the basis for the aforementioned protective factors. Likewise, the family can be one of the most important risk factors for alcohol or tobacco use initiation in adolescence, when adolescents have parents who use alcohol or tobacco, as well as when there is family breakdown and a poor relationship with parents.

In Brazil, cigarette advertising was banned in 2000. This contributed greatly to the reduction in the prevalence of smoking in the country. Studies have shown that adolescents who are widely exposed to these advertisements are the same ones who like them and who end up using these drugs frequently, that is, advertising does encourage consumption and contribute to the increase in the rates of all unfavorable outcomes of this consumption.^(^ ^7^ ^)^

There is considerable debate about the role of alternative (smokeless) tobacco products in reducing the harmful health effects of tobacco use. With regard to the use of such products, one should consider the Swedish experience with snus, a sublingual nicotine tablet adopted as an alternative to conventional cigarettes. A review on the use of snus suggests that it is less harmful to health than are cigarettes, reducing the risk of cardiovascular, respiratory and neoplastic diseases.^(^ ^11^ ^)^

Some of the global scientific community believes in the adoption of a regulatory policy that would discourage the use of the most harmful nicotine products^(^ ^12^ ^)^ (tobacco-burning cigarettes or products) in favor of products that are much less harmful, such as snus and, possibly, electronic cigarettes.^(^ ^13^ ^)^ Electronic cigarettes, because of their similarity to conventional cigarettes, have been shown to be the most attractive form of nicotine delivery to users, considering that worldwide consumption and sales have reached alarming figures in countries where their marketing is permitted. In Brazil, their marketing is prohibited. In the USA, this is already the preferred form of experimentation of 25% of young Americans.

The fact is that human frailty makes us vulnerable to using substances with psychoactive effects and that only cumulative scientific evidence allows the choice of public policies that can protect society from vulnerability and make life in society a reasonable bargain without it being degraded.

A society built upon a model of reckless and irrational consumption is, of course, vulnerable to drug use and the whole burden of this condition. Only a comprehensive, detailed analysis of the issue will allow the adoption of public policies that can minimize the impact of drug use and that are effective in reducing risk exposure, considering the vulnerability of the human condition.

