# Supplementary material for: Much more than cigarette smoking
Source: J Bras Pneumol. 2014 Mar-Apr;40(2):99–101. doi: 10.1590/S1806-37132014000200001 (PMC4083638; doi:10.1590/S1806-37132014000200001)
Supplement: Translation

# Translation: Muito além do tabaco

**Authors:** Jaqueline Scholz Issa, Gabriel Magalhães Lopes

**Affiliations:** Programa de Tratamento do Tabagismo, Departamento de Cardiopneumologia, Instituto do Coração, Hospital das Clínicas, Faculdade de Medicina, Universidade de São Paulo, São Paulo (SP) Brasil; Serviço de Psiquiatria da Infância e Adolescência e Programa Equilíbrio, Departamento e Instituto de Psiquiatria, Hospital das Clínicas, Faculdade de Medicina, Universidade de São Paulo, São Paulo (SP) Brasil

A condição humana fragilizada pelo medo da morte, pelo sofrimento afetivo, pelas doenças e pelo envelhecimento imprime ao ser humano a busca pelo prazer. O uso de drogas lícitas ou ilícitas é bom exemplo desse fato. Muitas vezes, isso ocorre de forma tão intensa que condena o indivíduo a morte prematura ou a subjugar a própria vida. Para agravar esta situação a sociedade ocidental, a todo o momento, é bombardeada por propagandas que incentivam o consumo de produtos. O consumo torna-se sinônimo de felicidade.

A indústria do tabaco talvez tenha sido a pioneira em explorar e abordar esse aspecto na publicidade do cigarro,^(^ ^1^ ^)^ sendo responsável pela indução de bilhões de usuários a dependência ao produto e, como consequência, a morte precoce e a perda de qualidade de vida, determinando um ônus sem precedentes aos sistemas de saúde ao redor de todo o planeta. A consequência dessa epidemia tabágica foi a adoção de medidas restritivas para reduzir o consumo, e isso motivou a indústria do tabaco a buscar produtos que pudessem manter a dependência a nicotina, mas com um rótulo como "menos prejudiciais à saúde" do que cigarros típicos.^(^ ^2^ ^)^ Dentro desse contexto, produtos como *hooka* ou narguilé, e mais recentemente o cigarro eletrônico, passaram a ser propagados como "inócuos", sem de fato terem respaldo científico para tanto. A evidência da presença de menos substâncias tóxicas nessas formas alternativas do tabaco em comparação ao cigarro convencional tem sido o argumento usado e explorado para isso, mas, de fato, não existem dados suficientes para se considerar que o uso dessas substâncias em menor concentração, mas de forma contínua e perpetuada pela presença do vapor de nicotina, seja inócuo.

No epicentro dessa discussão, o presente volume do Jornal Brasileiro de Pneumologia apresenta o artigo original de Martins et al.^(^ ^3^ ^)^ sobre a experimentação de narguilé, a prevalência de seu uso e o conhecimento sobre o assunto entre estudantes de medicina do terceiro e sexto anos da Faculdade de Medicina da Universidade de São Paulo, em São Paulo (SP), entre 2008 e 2013. Apesar de a maioria dos estudantes reconhecer o potencial de risco a saúde pelo uso do produto, seja pelo risco de contaminação por bactérias e vírus, seja pelo próprio potencial de dependência a nicotina determinado pelo uso do produto, além da alta exposição a elevadas concentrações de monóxido de carbono, tal conhecimento não impediu que mais de 40% daqueles estudantes do sexo masculino experimentassem o produto. A baixa prevalência do uso de cigarros entre os estudantes de medicina do terceiro e sexto anos de curso (9,78% e 5,26% entre os homens, respectivamente, e 1,43% e 2,65% entre as mulheres) claramente demonstra a efetividade das políticas públicas de desestímulo à iniciação do tabagismo, realizadas há mais de 20 anos no Brasil; por outro lado, isso reforça o alcance do marketing e da publicidade que disseminam o uso de formas alternativas do tabaco.

A redução do tabagismo no Brasil nos últimos 5 anos tem sido gradual e constante entre os homens, com uma variação de 21% em 2008 para 18% em 2012.^(^ ^4^ ^)^ Essa taxa entre as mulheres diminuiu e tem se mantido estável desde 2008, oscilando entre 12% e 13%. Atualmente, já temos mais ex-fumantes que fumantes no Brasil e eles representam 22% da população. Em uma revisão sistemática da literatura sobre o uso de álcool e tabaco entre adolescentes de 10 a 19 anos, Barbosa et al.^(^ ^5^ ^)^ encontraram que a prevalência de uso atual de tabaco (uso na época da pesquisa ou no mês anterior) variou de 2,4% a 22,0%, com média de 9,3% no Brasil. Embora o risco de início de consumo de tabaco por adolescentes seja maior quando têm pais fumantes, há outros fatores familiares associados, como práticas parentais com baixa aceitação e baixo controle comportamental dos filhos.^(^ ^6^ ^)^ Além das implicações do uso do tabaco sobre a saúde do adolescente, há que se ressaltar também que seu uso associado ao consumo de maconha é elevado.

A indústria do tabaco sabe que tem ao seu lado fatores que facilitam por demais a experimentação de qualquer produto que contenha nicotina entre os jovens, primeiramente pela curiosidade, assim como pela influência da simbologia de ritual de passagem, ausência de maturidade de estruturas cerebrais que inibem atos de impulsividade, influência do grupo e o próprio desejo de enfrentamento, ou seja, um conjunto de fatores que deixa os jovens particularmente seduzidos pelo fascínio de experimentar e consumir drogas.

É relevante destacar a importância da influência cultural e da mídia sobre os jovens, visto que a adolescência é uma fase crucial no desenvolvimento da personalidade e da individualidade. Nessa fase, o adolescente é muito influenciável, e o marketing e a propaganda atingem propositalmente os jovens, os tendo como alvos. Estudos evidenciam que a propaganda de cigarros tem uma grande influência no consumo de tabaco por jovens.^(^ ^7^ ^)^ A divulgação via judicial de documentos secretos da indústria do tabaco nos EUA comprova a estratégia de direcionar a propaganda do cigarro preferencialmente para crianças e adolescentes.

O resultado é que as novas vivências tendem a ser experimentadas com grande intensidade, e o uso de álcool, cigarros e outras drogas é um comportamento frequente, o que torna esse período de vida crucial na maioria dos estudos e programas de prevenção de dependência de substâncias. Aproximadamente 72% dos adolescentes nos EUA relatam ter experimentado álcool; no Brasil, essa prevalência chega a 84,3% entre estudantes de 17-18 anos de idade.^(^ ^8^ ^)^ Em relação aos cigarros, nos EUA, a prevalência de seu uso alguma vez na vida é de 43,6% entre adolescentes e, no Brasil, essa prevalência é de 32,1% aos 18 anos.

Em 2009, segundo a Pesquisa Nacional de Saúde do Escolar,^(^ ^9^ ^)^ 24,2% dos escolares que frequentavam o nono ano do ensino fundamental experimentaram cigarros e 6,3% os usavam habitualmente. Não houve diferenças entre meninos e meninas; no entanto, o uso foi maior nas escolas públicas (26,4%) do que nas escolas privadas (18,3%).

Os fatores ambientais constituem importantes fatores de risco para a experimentação e a manutenção do uso de álcool e tabaco na adolescência, bem como a progressão para outras drogas.^(^ ^10^ ^)^ Os fatores de proteção são menos pesquisados na literatura do que os fatores de risco. Os principais fatores de proteção relativos ao uso de álcool e cigarros são a capacidade de enfrentar e superar problemas, principalmente no sexo feminino; a religiosidade; a realização de pelo menos uma refeição com pais ou responsáveis na maioria dos dias da semana; e o conhecimento dos pais ou responsáveis sobre o que os adolescentes fazem em seu tempo livre nos últimos 30 dias. O monitoramento parental eficaz parece ser o maior fator de proteção ao uso de álcool, tabaco e outras drogas na adolescência e pode ser a base para os fatores de proteção supracitados. Da mesma forma, a família também pode ser um dos fatores de risco mais importantes para o início de uso de álcool ou tabaco na adolescência, quando os adolescentes têm pais que também fazem uso de álcool ou de tabaco, assim como quando há desestruturação familiar e relacionamento ruim com os pais.

A propaganda do cigarro foi proibida no Brasil em 2000. Esse fato contribuiu sobremaneira para a redução na prevalência do tabagismo no país. Estudos mostram que os adolescentes que são mais expostos a essas propagandas são os mesmos que as apreciam e que acabam fazendo uso mais frequente dessas drogas, ou seja, a propaganda comprovadamente estimula o consumo e contribui para o aumento de todos os desfechos desfavoráveis desse consumo.^(^ ^7^ ^)^

Há um debate considerável sobre o papel dos produtos alternativos ao tabaco (*smokeless*) em reduzir os danos à saúde causados pelo tabaco. Dos produtos alternativos ao tabaco, a Suécia tem experiência com uso do *snus*, um tablete de nicotina de uso sublingual adotado como alternativa ao cigarro comum. Uma revisão sobre uso de *snus* sugere que ele é menos prejudicial para a saúde do que cigarros, reduzindo o risco de doenças cardiovasculares, respiratórias e neoplásicas.^(^ ^11^ ^)^

Parte da comunidade científica mundial acredita na adoção de uma política de regulamentação que determine o desestímulo ao consumo dos produtos de nicotina mais prejudiciais^(^ ^12^ ^)^ (cigarros ou produtos de tabaco inalado por combustão) por produtos que são muito menos prejudiciais, como o *snus* e, possivelmente, o cigarro eletrônico.^(^ ^13^ ^)^ Esse último, devido à semelhança com o cigarro convencional, tem se mostrado a forma mais atrativa de consumo de nicotina, considerando que o consumo e venda mundial têm alcançado cifras alarmantes nos países onde sua comercialização é permitida. No Brasil, a comercialização do produto é proibida. Nos EUA, essa já é forma de experimentação preferida por 25% dos jovens americanos.

O fato é que a fragilidade humana nos torna vulneráveis ao uso de substâncias com efeitos psicoativos e somente as evidências científicas cumulativas permitem a escolha de políticas públicas que possam proteger a sociedade da vulnerabilidade e compatibilizar a vida em sociedade sem degradá-la.

Uma sociedade construída num modelo de consumo inconsequente e irracional evidentemente fica vulnerável ao consumo de drogas e a todo ônus dessa condição. Somente a análise abrangente e criteriosa da questão permitirá adoção de políticas publicas que minimizem o impacto do consumo de drogas e que sejam efetivas em reduzir a exposição ao risco, considerando a vulnerabilidade da condição humana.
